# Detecting outliers in case-control cohorts for improving deep learning networks on Schizophrenia prediction

**DOI:** 10.1515/jib-2023-0042

**Published:** 2024-07-15

**Authors:** Daniel Martins, Maryam Abbasi, Conceição Egas, Joel P. Arrais

**Affiliations:** Centre for Informatics and Systems, Department of Informatics Engineering, University of Coimbra, Coimbra, Portugal; Polytechnic Institute of Coimbra, Applied Research Institute, Coimbra, Portugal; Research Centre for Natural Resources Environment and Society, Polytechnic Institute of Coimbra, Coimbra, Portugal; Centre for Innovative Biomedicine and Biotechnology, University of Coimbra, Coimbra, Portugal; Biocant – Transfer Technology Association, Cantanhede, Portugal

**Keywords:** machine learning, deep learning, phenotype prediction, Schizophrenia

## Abstract

This study delves into the intricate genetic and clinical aspects of Schizophrenia, a complex mental disorder with uncertain etiology. Deep Learning (DL) holds promise for analyzing large genomic datasets to uncover new risk factors. However, based on reports of non-negligible misdiagnosis rates for SCZ, case-control cohorts may contain outlying genetic profiles, hindering compelling performances of classification models. The research employed a case-control dataset sourced from the Swedish populace. A gene-annotation-based DL architecture was developed and employed in two stages. First, the model was trained on the entire dataset to highlight differences between cases and controls. Then, samples likely to be misclassified were excluded, and the model was retrained on the refined dataset for performance evaluation. The results indicate that SCZ prevalence and misdiagnosis rates can affect case-control cohorts, potentially compromising future studies reliant on such datasets. However, by detecting and filtering outliers, the study demonstrates the feasibility of adapting DL methodologies to large-scale biological problems, producing results more aligned with existing heritability estimates for SCZ. This approach not only advances the comprehension of the genetic background of SCZ but also opens doors for adapting DL techniques in complex research for precision medicine in mental health.

## 1 Introduction

Complex diseases are marked by an intricate interplay of genetic, environmental, and physiological factors [1]. Grasping the underlying mechanisms of complex diseases demands a multifaceted approach encompassing genomics, epidemiology, bioinformatics, and clinical research, accompanying the advent of personalized medicine [2].

Schizophrenia (SCZ) stands as an example of such conditions. Several variables converge to give rise to a chronic and severely disabling mental illness characterized by an array of cognitive, emotional, and perceptual disturbances [3]. Despite family and twin studies consistently underscore a substantial heritable component [4, 5], the precise etiology of SCZ remains elusive [6]. Several studies over the years have identified risk genes associated with dopamine, glutamate and GABAergic systems [7, 8]. Nonetheless, its specific role and degrees of contribution to the disease onset are unclear.

The Diagnostic and Statistical Manual of Mental Disorders (DSM-5), published by the American Psychiatric Association in 2013 [9], presents specific criteria for SCZ diagnosis, including hallucinations, delusions, social withdrawal, and cognitive impairments. However, these criteria still yield a broad framework, as the clinical presentation of SCZ varies widely among individuals. Furthermore, the diagnosis of SCZ is hindered by its overlap with other psychiatric disorders [10]. Nonetheless, the prevalence of SCZ (0.32 % according to the WHO [11]) has consistently increased over the years. Particularly within the working-age demographic in the most developed countries [3], and in regions situated at higher latitudes [12].

Due to its recurrently reported misdiagnosis rates [13–15], SCZ is likely to pose a significant challenge for studies focused on achieving a more detailed understanding of its general genetic foundations. Scandinavian countries have been proficient in analyzing that problem, and extensive case-control cohorts from these regions have been drawn and studied under the scope of SCZ [16–20]. These cohorts benefit from a historical prevalence of a more conservative diagnosis in this region. This diagnostic approach is majorly focused on the biological component of SCZ etiology [17, 21].

Swedish cases are typically identified and selected from the Hospital Discharge Register (HDR), established in 1964 and covering all psychiatric clinical diagnoses in the country since 1987 [22]. Initial investigations into the accuracy of registered SCZ diagnoses revealed relatively strong agreement, ranging from 76 % to 81 %, when compared to reevaluations of the cases using the criteria outlined in the DSM-III [21], in 1986 [23], and the DSM-IV [22, 24], in 1994 [25]. A notably higher concordance rate of 94 % emerged when considering a broader spectrum of diagnoses encompassing schizophrenic psychoses, such as SCZ, schizoaffective psychosis, or schizophreniform disorder [16]. Consequently, the HDR proved a dependable criterion for defining cases under this broader classification. However, when focusing exclusively on SCZ diagnoses, the concordance rate remained consistent with previous reports at 75 %.

Despite these limitations, the underlying influence of genetic factors on SCZ has been intensively studied over time. In recent years, the implementation of Machine learning (ML) [26] and Deep Learning (DL) [27] architectures on this problem has gained relevance. To our knowledge, the best predictability for SCZ achieved up to this point, when solely addressing genomics data, derived from the application of the GenNet Framework, presenting maximum AUC values of 0.73–0.74 [28]. The original work estimates upper bounds for the accuracy of a classification model, depending on three factors: the distribution of cases and controls, the positive diagnosis concordance rate on monozygotic twins, and the prevalence of the disease. For the SCZ dataset used for their work, that upper bound would vary between 0.73 and 0.83, according to the minimum and maximum monozygotic twins concordance considered from literature (41–65 %) [5, 29]. As a result, although this perspective suggests a defined mark for the performance of models addressing purely genetic information, it still presents a range of results that would depend on additional information. The present study will attempt to understand to what extent the misdiagnosis rate on SCZ could contribute to the referred result variability by employing a sample filtering procedure that relies on the misdiagnosis rates reported in the literature.

This work attempts to optimize the application of DL Architectures to a biological problem, taking advantage of prior knowledge on different scopes. Firstly, by applying efficient pre-processing, reducing the number of features to the variants more likely to present a contribution to the phenotype manifestation. Secondly, by designing a network based on reference genetic annotations, and lastly, by analyzing the impact of outliers on the model performance.

## 2 Workflow

**Figure 1:**
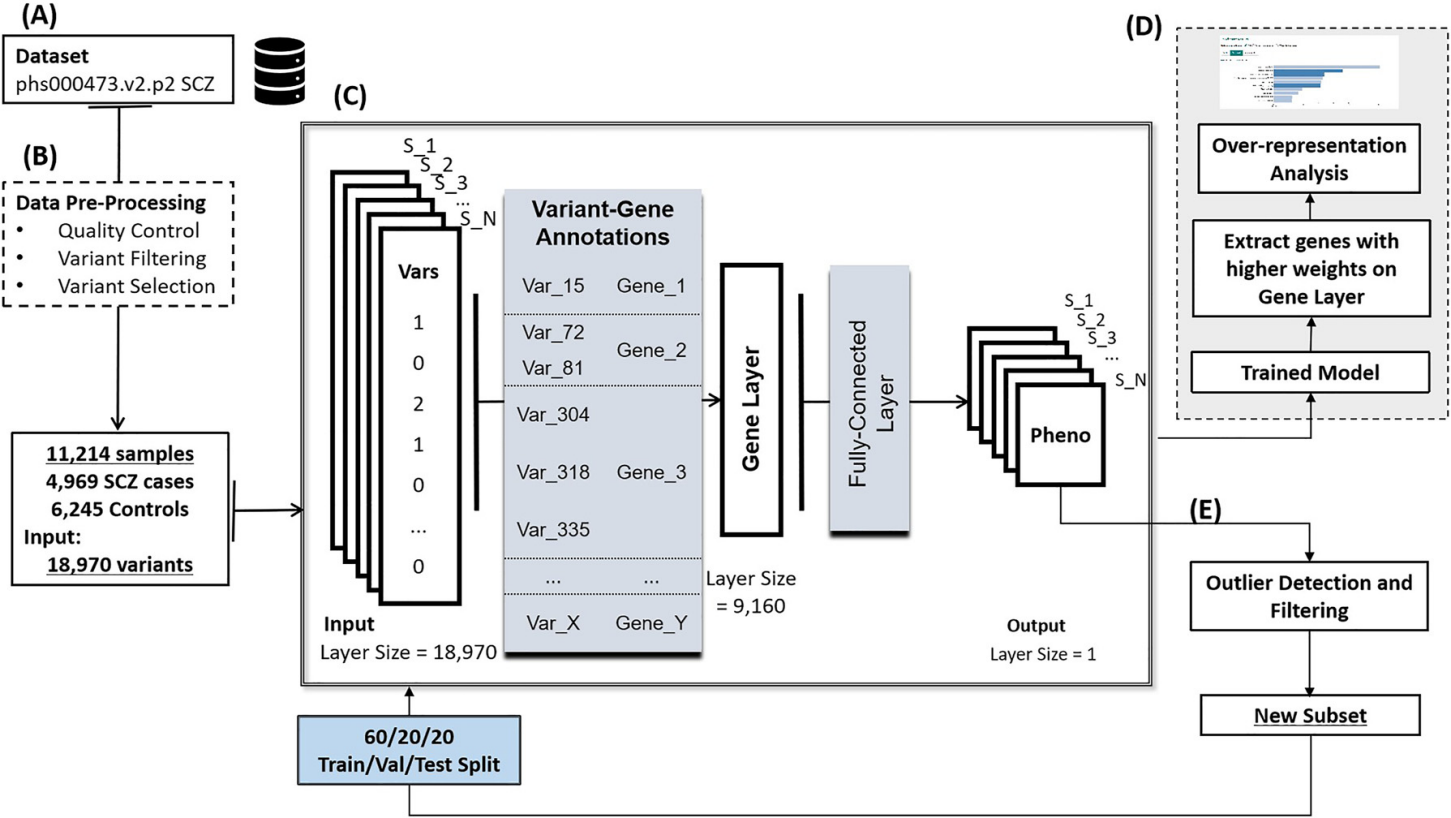
General workflow of the proposed model. (A) Input dataset: SCZ and bipolar disease, (B) preprocessing step, (C) deep model architecture, (D) over-representation analyses, (E) sample filtering.

The present study was designed to analyze a reduced, yet representative, variant subset of an input Whole-Exome Sequencing (WES) dataset, detect a basal phenotype-driven distinction of the samples and then, gauge the influence of outlying samples on the overall performance of a ML model. Figure 1 illustrates the overall workflow of the study. We utilized a WES dataset based in Sweden, which is stored in the Genotype and Phenotypes (dbGaP) database (1.A). This dataset was subjected to stringent Quality Control measures to retain only the most relevant variants (1.B). The resulting dataset, comprising 18,970 variants across 11,214 samples, was then employed as input for a robust deep neural network architecture developed using the Keras-based GenNet framework (1.C) [28], which was used to train the classification models.

The network comprised three pivotal layers: the input layer for genetic variants, encoded by quantifying the number of alternative alleles in the genotype; an intermediary gene layer; and the output layer for phenotypic predictions. The framework relied on a thoughtfully created file that held crucial links between genetic variants and genes. This file played a significant role in defining the connections within the network, resembling a complex web of biological interactions. This model was trained for two distinct stages of the study. Firstly, using the entirety of the dataset. The weights of the gene and the output layer from the model trained under this condition were employed to conduct, respectively, Pathways Over-Representation Analyses (1.D) and an outlier filtering procedure (1.E).

On this step, we exclude the cases with the lowest scores by a proportion based on literature-reported misdiagnosis rates, resulting in subsets of cases and controls that were used, on the second stage of the study, as input for new instances of the model. Trained, validated and tested with conventional splits of the input data.

### 2.1 Dataset Schizophrenia

In this study, we utilized the *Sweden-Schizophrenia Population-Based Case-Control Exome Sequencing* dataset archived with the accession code phs000473.v2.p2 from the dbGaP database (Figure 1.A) [30]. This dataset comprises a total of 12,380 samples, including 6,245 controls and 6,135 cases. It reports the information for 1,811,204 variants identified in, at least, one sample. Among the cases, 4,969 samples report to a SCZ diagnosis, and 1,166 to bipolar disease.

The SCZ cases were originally identified from the Swedish Hospital Discharge Register, ensuring that they had at least two hospitalizations with a discharge diagnosis of SCZ. The persistence of any medical or psychiatric disorder records that could potentially affect a reliable SCZ diagnosis served as an additional exclusion criterion for this dataset. Controls were randomly selected from general population registers, being excluded by any register of hospitalizations for SCZ. Additionally, both SCZ cases and controls in the study were required to be at least 18 years old, and both of their parents born in Scandinavia.

### 2.2 Data preprocessing

The original dataset had already undergone a filtering step based on Phred-score quality (*QUAL*) [31] for variant calls, which retained only variants with *QUAL* > 30. Further filtering steps were followed to ensure the quality of the data and select the information to use as input for subsequent steps (Figure 1.B).

#### 2.2.1 Quality control and filtering

As in the majority of previous studies on this dataset [17–19], only SCZ cases were considered for this work. We excluded bipolar samples from the dataset using BCFtools (version 1.17) [32]. Hereupon, the present work relies on 6,245 controls and 4,969 SCZ cases.

Subsequently, we recalculated the metrics for each variant site using the BCFtools plugin called *fill-tags*. Variants with a recalculated mean read depth (*DP*) below eight or a genotype call rate below 90 % were further excluded from the dataset, employing VCFTools (Version 0.1.15) [33]. To address multi-allelic variants in the VCF file, we used BCFtools to separate them and set as *missing* all genotypes that included an allele different to the reference (REF) or the alternative (ALT) in the new record. The split variant records were then filtered based on the previously presented criteria for its recalculated mean read depths and call rates, ensuring that only the variants with enough information for the two major alleles were kept.

To further grant the quality of the variant calls on the dataset, the Variant Quality Scores Recalibration steps outlined in the Genome Analysis Toolkit Best Practices Workflow (Version 3.8) were followed [34]. A total of 1,199,689 variants passed all quality control and filtering procedures.

#### 2.2.2 Variant selection with association test

At this point, the number of features was greater than the number of samples by 2 orders of magnitude (1,199,689 variants for 11,214 samples), raising a dimensionality problem for ML [35].

To reduce the dimensionality of the dataset while maintainig the variants with greater relevance for case-control distinction, a chi-squared test was conducted on all variants, using a 3 × 3 contingency table to count the three possible genotypes among cases and controls. To homogenize the genetic variables under investigation, InDels and variants located on sexual chromosomes were filtered out, thus remaining 1,142,236 SNPs. Among those, 18,970 SNPs presented significant associations.

The identified SNPs were annotated using the most recent available Annovar version, referent to the hg19 genome build as of October 19, 2021 [36]. We chose not to adjust the *p*-values obtained from the chi-squared test for multiple comparisons. This deliberate choice, while reducing the dimension of the dataset, would allow us to maintain the representativity of the dataset. To assess this representativity, an over-representation analysis will be performed on the classification model results.

After the preprocessing steps (Figure 1.B), the dataset comprised a total of 18,970 variants located on 9,160 distinct genes, serving as input for the model (Figure 1.C).

### 2.3 Deep neural network

We used a neural network framework built on Keras to create the model (Figure 1.C), which consists of three layers: input (representing variants), gene, and output (for phenotype prediction). To represent variants, we encoded them by counting the number of alternative alleles. This process is used to transform the raw genetic data into a discrete format that the neural network can effectively process and learn from (Table 1).

**Table 1:** Genotype representation and encoding following an additive model. Under this approach, alternative alleles on each genotype are counted in order to represent data with discrete values.

Genotype	Representation	Encoding
Homozygous for reference allele	0/0	0
Heterozygous	0/1	1
Homozygous for alternative allele	1/1	2

The framework requires a “topology” file that contains information about the associations between variants and genes, which we obtained from Annovar annotation [36]. The file format is presented in Table 2. This file has *x* rows, where *x* corresponds to the number of genetic variants used as input features in this study, which is 18,970. Each line in the topology file provides information about the gene associated with a specific variant. Consequently, the number of genes (*y*) is less than or equal to the number of variants (*x*), and in this study, we considered 9,160 genes.

**Table 2:** Exemplification of a *topology* file used as input on the Gennet framework. Variant-gene associations, annotated from ANNOVAR, will define the model connections between the input and the gene layer. Each input (variant) layer node is uniquely connected to one gene layer node, gene layer nodes are connected to one or more input (variant) layer node. For this study, *x* = 18, 970 and *y* = 9, 160.

Variant node	Variant	Gene node	Gene
0	Variant 0	0	Gene 0
1	Variant 1	0	Gene 0
2	Variant 2	0	Gene 0
3	Variant 3	1	Gene 1
…			
*x* − 2	Variant *x* − 2	*y* − 1	Gene *y* − 1
*x* − 1	Variant *x* − 1	*y*	Gene *y*
*x*	Variant *x*	*y*	Gene *y*

The framework we used employs a knowledge-driven interlayer connection. The information in the topology file is crucial for building the network in the model. It defines the connections between the input (variant) layer, which has 18,970 nodes, and the gene layer, which has 9,160 nodes. Each connection is determined by the associations provided in each row of the file. This means that each node in the input layer is connected to one node in the gene layer, and each node in the gene layer is fed by one or more nodes from the input layer. In the end, the gene layer is fully connected to the output layer to complete the model.

This neural network architecture ensures that the weights associated with variants are organized into their corresponding genes. In doing so, it establishes an interaction hierarchy reminiscent of biological models. This hierarchy operates in two ways: first, by aggregating the combined effects of variants on the gene, and second, by considering the impact of gene alterations on the organism’s phenotypes. The sequential nature of the network implies that stronger weights will contribute proportionally more to the final phenotype predictions. It’s important to note that these weights do not represent statistical correlations with the phenotype. Instead, they reflect the relative importance of each gene or variant in distinguishing the genetic profiles between the cases and controls provided as input.

In this network, the gene weights correspond to parallel regressions of their respective variant weights. Additionally, the gene layer serves as the coefficients for a final logistic regression to calculate the ultimate output.

All models trained for this work used a batch size of 64 and were optimized with the Adam optimizer and a binary cross-entropy loss. During training, this loss function penalizes wrong predictions and subsequently, the weights of the network nodes are adjusted to minimize the total loss, and thus, maximize the distinction between cases and controls.

### 2.4 Sample filtering

To evaluate the potential impact of misclassified samples on our model’s performance, we first need to identify such samples. It’s essential to clarify that the term “misclassified” does not imply a clinical diagnosis, but rather indicates that a particular sample exhibited a genetic profile that deviated from its expected class. Within this work, the term “misclassified” refers to model predictions and “misdiagnosis rate” refers to the clinical estimatives cited from the literature.

Genetic data alone lacks empirical support for robust selection criteria. Therefore, we rely on dataset-driven outlier detection. Given two critical factors: (i) the strong genetic influence on phenotype manifestation and (ii) a consistent but minority count of misclassified samples, our approach involves training the model on the entire dataset. This strategy encourages the model to distinguish genetic profiles between cases and controls, while favoring prevalent features within each group. Consequently, in the dataset, misclassified samples are more likely to exhibit distinct genetic profiles compared to the majority within their respective classes (case or control).

To improve the ability to differentiate between specific sub-categories within both the case and control groups, we conducted training and testing on the complete dataset. Early stopping was enforced after 200 epochs. Training the model on the entire dataset served as an outlier detection method (Figure 1.E), with a focus on identifying both cases and controls with similar genetic profiles.

Our analysis involved calculating a simple average of the test classification scores for ten independent training instances, with predictions ranging from 0 to 1 without binary conversion. Subsequently, we excluded a subset of misclassified controls and cases, retaining those with the highest and lowest scores, respectively, for further investigation.

The proportion of samples removed from the control subset was determined based on the reported prevalence of SCZ in Sweden, as documented by the Gillberg Neuropsychiatry Centre at the University of Gothenburg (0.34 %) [37].

For the case subset, the proportion of samples removed was determined by considering the lowest SCZ misdiagnosis rates in Sweden, accounting for both the broad (6 %) and narrow (19 %) definitions of SCZ, as reported in literature [16, 21, 22, 24]. Following the final model training, we compared the average test Area Under the Curve (AUC) scores using a two-tailed unpaired *t*-test.

### 2.5 Pathway and gene-disease analysis

Pathway enrichment analysis plays a crucial role in providing mechanistic insights into gene lists generated from genome-scale (omics) experiments. This method identifies biological pathways that exhibit a higher level of enrichment within a gene list than would be expected by random chance [38]. For this work, enrichment analyses were performed on the genes prioritized by our model to evaluate their association with pathways previously linked to SCZ in the literature and thus, assess the representativity of the dataset (Figure 1.D). We extracted the genes corresponding to nodes with higher weights and performed an over-representation analysis using the 2019 online edition of WebGestalt (WEB-based GEne SeT AnaLysis Toolkit) [39]. These analyses were performed against PANTHER (Protein Analysis Through Evolutionary Relationships) v3.6.1 [40, 41], KEGG (Kyoto Encyclopedia of Genes and Genomes) Release 88.2 [42], and OMIM (Online Mendelian Inheritance in Man) [43]. The entire genome served as the reference set.

## 3 Application

In the following section, we examine the experimental analysis and present the findings derived from this study. This research endeavor contained a multi-phase approach, incorporating feature selection, the application of advanced DL methodologies to detect outlying samples within the dataset, and the development of a robust classification model. Each step contributed to understanding the genetic factors associated with SCZ and helped refine the model’s performance.

### 3.1 Feature selection

We began by working with a substantial dataset comprising 1,811,204 variant sites. To ensure data quality and relevance, we performed several filtering steps. After excluding variants in sexual chromosomes, InDels, and variants that did not retain any altered genotype after the exclusion of Bipolar disease case samples, we were left with a working dataset containing 1,142,326 variants.

**Figure 2:**
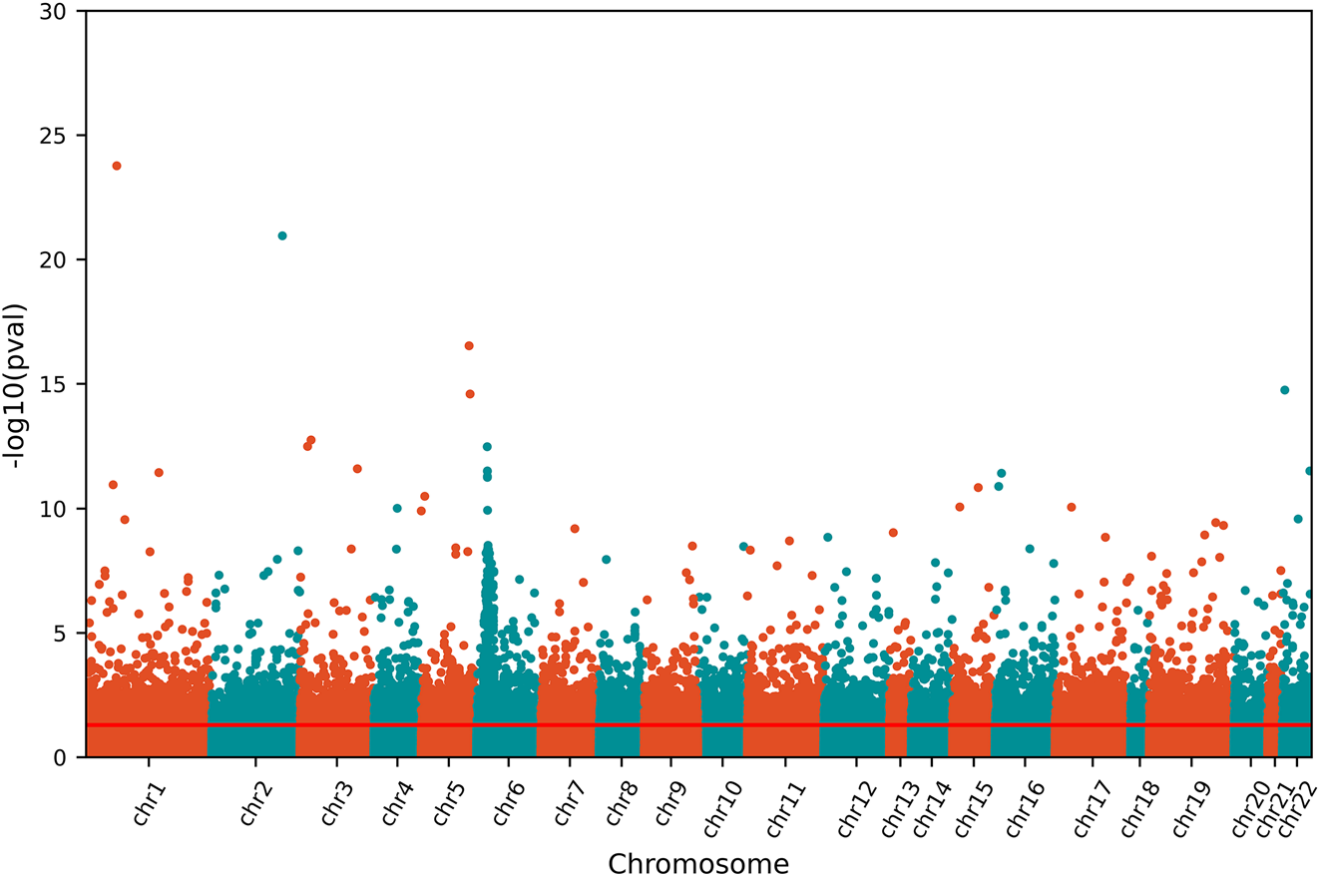
Manhattan plot for genotype-phenotype associations within the tested SNPs. The *p*-values for chi-squared tests on 3 × 3 contingency tables for each variant are presented as the negative of its common logarithm. 18,970 variants present a significant association, defined by *p*-value <0.05 .

Using a chi-squared test, we identified a total of 18,970 autosomal Single Nucleotide Polymorphisms (SNPs) that exhibited a statistically significant association with SCZ (Figure 2). Importantly, we did not apply corrections to the *p*-values for genome-wide significance at this stage, as our primary goal was to reduce the number of features while maintaining the representativeness of the data.

The reduced dataset accounts for roughly 1 % of the initial data. Within this subset, 8,971 variants, equivalent to 47.3 % of the dataset, had an allele frequency (AF) of less than 1 %, 2,695 variants, accounting for 14.2 %, had an AF between 1 and 5 %, while 7,304 variants, constituting 38.5 %, had an AF of 5 % or greater. It’s noteworthy that there were no singletons identified in this specific subset.

### 3.2 Application of deep network for detection of sampling outliers

To explore the genetic diversity within both the SCZ cases and control groups, we employed DL techniques. This process served to emphasize the internal variations within the trained dataset, allowing us to better highlight any inherent differences between the misclassified samples and either cases or controls.

Our DL models underwent training and testing on the complete dataset, achieving an average test Area Under the Curve (AUC) score of 0.9241 with a standard deviation of 0.0014 (95 % confidence interval [0.923, 0.925]). We observed that 2.7 % of all controls and 7.8 % of all cases were consistently misclassified.

To investigate whether the differentiation between cases and controls was primarily influenced by genetic factors related to SCZ, we calculated the mean weights for each node in the gene layer across the ten models. Since the models are trained to maximize the distinction between cases and controls by increasing the weights of nodes with greater influence to the output, the nodes with higher weights on the gene layer are expected to be referent to genes associated to SCZ. We selected the top 150 genes with the highest mean scores for subsequent functional analysis.

Our analysis revealed significant over-representations in pathways previously related to SCZ in the literature, with a special emphasis on the instances that maintained that significance after controlling for the False Discovery Rate (FDR). The beta-adrenergic signaling pathways showed significant enrichment when analyzed with the PANTHER reference set. When analyzed with KEGG, we observed significant associations with pathways related to Extracellular Matrix receptors and cardiomyopathy. Lastly, we detected a significant over-representation of genes associated with SCZ when analyzed with the OMIM database (Table 3).

**Table 3:** Over-representation analyses on WebGestAlt with PANTHER, KEGG and OMIM data. Enrichment ratios quantify the over-representation of genes associated to a given pathway within a provided gene list in comparison to the same proportion within a reference set, for this study, the entire genome was used as the reference set.

	Enr. R.	*p*-value	FDR	Ref.
**PANTHER**				
Beta1 adrenergic receptor sign. path.	9.6	6.4 × 10^−4^	3.6 × 10^−2^	[56]
Beta2 adrenergic receptor sign. path.	9.6	6.4 × 10^−4^	3.6 × 10^−2^	[56]
**KEGG**				
ECM-receptor interaction	8.4	2.9 × 10^−4^	3.3 × 10^−2^	[57]
Hypertrophic cardiomyopathy	8.3	3.1 × 10^−4^	3.3 × 10^−2^	[58]
Dilated cardiomyopathy	7.7	4.5 × 10^−4^	3.7 × 10^−2^	[59]
**OMIM**				
SCZ	57.4	5.0 × 10^−4^	2.5 × 10^−2^	

Enr. R., enrichment ratio; FDR, correction for false discovery rate; Ref., reference for literature association to SCZ; sign. path, signaling pathway.

Complementarily, there were also identified associations with Tetrahydrofolate biosynthesis (*p*-value = 4.7 × 10^−2^, FDR = 0.77) [44], Oxytocin (1.4 × 10^−2^, 0.39) [45], 5-HT2 (2.1 × 10^−2^, 0.39) [46] and Nicotinic acetylcholine (1.1 × 10^−2^, 0.39) [47] receptors signaling pathways and the integrin signaling pathway (1.9 × 10^−2^, 0.39) [48] against the PANTHER reference set; the calcium signaling pathway (1.9 × 10^−3^, 9.0 × 10^−2^) [49], focal adhesion (2.9 × 10^−3^, 0.12) [50] and the PI3K-Akt signaling pathway (3.6 × 10^−3^, 0.13) [51] against the KEGG reference set; and Osteoporosis (1.5 × 10^−2^, 0.25) [52], Prostate (3.2 × 10^−2^, 0.35) [53] and colorectal cancer (3.4 × 10^−2^, 0.35) [54] and susceptibility to HIV Type 1 (3.7 × 10^−2^, 0.35) [55] against the OMIM database. However, despite being previously linked with SCZ in the literature, all those associations failed the control for FDR and must be considered with caution.

These results indicate that the distinction between model classes is influenced by the weights assigned to SCZ-related genes. Therefore, cases and controls that are misclassified by the model are more likely to have genetic profiles that differ from the typical SCZ-related genetic patterns.

Following this assessment on the training data, we computed and arranged the mean test scores for both the cases and controls. Since test scores under 0.5 correspond to a classification of *control* and test scores above or equal to 0.5 correspond to a classification of *case*, the *true* controls with higher scores and the *true* cases with lower scores were removed for the next procedures. In order to align with the documented prevalence of SCZ in Sweden, which stands at 0.34 % [37], we removed the 21 control samples with the highest mean scores.

As for the cases, we aimed to establish a threshold that corresponded to the lowest reported misdiagnosis rate (6 %) for a broad definition of SCZ in Sweden [16]. This led to the exclusion of the 300 cases with the lowest mean scores. The mean score of the last removed sample in this category was 0.30.

Additionally, we considered the scenario reflecting the lowest misdiagnosis rate (19 %) reported in Sweden for a narrower definition of SCZ [21]. In this case, there were only considered for exclusion the samples misclassified in at least half of the tests. These criteria lead to the removal of 944 cases. The mean score of the last sample removed under this criterion was 0.41.

**Figure 3:**
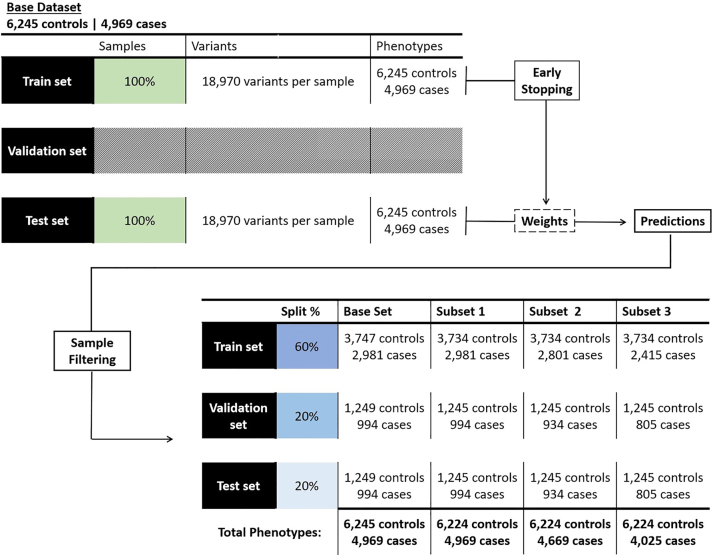
Sample filtering and split of new subsets for training new instances of the model. After training the model with the entire data, the weights on the output layer, for each sample, are used to select the outlying samples to remove on further analyses. Posteriorly, all subsets are split in the same proportions for training, validating and testing the new instances of the model.

The subsets selection and design process is depicted on Figure 3.

### 3.3 Model classification performance on filtered subsets

To evaluate the performance of the classification model, we divided the input dataset, containing 18,970 variants. Samples were split into training (60 %), validation (20 %), and test (20 %) sets, maintaining the proportion of cases and controls in each set. This process was repeated thirty times for each subset under analysis, ensuring robustness. Prior to each run, the samples underwent complete randomization at the splitting step.

Our benchmark metrics were obtained from the GenNet original publication [28]. Both the benchmark and our base model were trained using the entire dataset, comprising 4,969 cases and 6,245 controls. Subsequently, we developed three models. The models (Model 1, Model 2, and Model 3) were trained on modified datasets that exclude specific samples based on our outlier detection and misclassification criteria.

Model 1 was trained with a modified dataset, excluding 21 misclassified controls. For model 2, there were excluded both misclassified controls and cases. The latter amount to 300 samples, following the misdiagnosis rate under the broader definition of SCZ. For Model 3, the same controls were excluded and the SCZ misdiagnosis rate for its narrower definition was employed, leading to the exclusion of 944 cases (Figure 3).

All models were trained and optimized using a batch size of 64 with the Adam optimizer and a binary cross-entropy loss.

Our analysis revealed that the average test AUC scores of Model 2 and Model 3 were significantly different to the results on the Base model, with *t*(18) = 8.77, *p* < 0.0001 and *t*(18) = 20.24, *p* < 0.0001, respectively. Furthermore, it also evidenced a significant distinction between Model 2 and Model 3 results, with *t*(18) = 14.82, *p* < 0.0001, indicating improved model performance on filtered data. Overall, Model 3, trained on a subset for which 944 cases (19 %) were excluded, present the most improved performances (Table 4).

**Table 4:** Performance of the tested models. The base model was trained with an unfiltered dataset. For model 1, 2 and 3, there were excluded 21 misclassified controls. In addition to that, for model 2, there were excluded 300 cases, corresponding to 6 % of the data on that class, and also the misdiagnosis rate under the broader definition of SCZ. And for Model 3, there were excluded 944 cases, corresponding to 19 % of the data on that class, and also the misdiagnosis rate under the narrow definition of SCZ. Avg. AUC – average for the area under the curve values; Max. AUC – maximum area under the curve value among the experiments considered.

Model	Validation		Test	
	Avg. AUC	Max. AUC	Avg. AUC	Max. AUC
GenNet (benchmark)	0.70 ± 0.018	0.73	0.72 ± 0.016	0.74
Base	0.71 ± 0.013	0.74	0.71 ± 0.012	0.73
Model 1	0.71 ± 0.011	0.73	0.71 ± 0.009	0.74
Model 2	0.75 ± 0.007	0.76	0.75 ± 0.008	0.76
Model 3	0.82 ± 0.010	0.84	0.81 ± 0.010	0.83

## 4 Discussion

As large-scale genetic studies became more prevalent, its potential for scientific research greatly increased. Scandinavian nations, with Sweden standing out in particular, epitomize this paradigm for SCZ, in particular. The largest Exome Sequencing case-control cohort on the disease was originated in that country, and it has been thoroughly analyzed over the years.

ML models offer fresh avenues for enhancing outcomes and exploring biological problems, leveraging the growing wealth of data over the years. Nonetheless, the effectiveness of classification models critically relies on a robust and dependable definition of classes in the training data. Most case-control studies contend with an inherent rate of dataset misclassification, hindering the direct adaptation of ML classification algorithms to address biological problems.

In the case of SCZ, this problem is accentuated by absence of clearly defined genetic and biochemical biomarkers [60]. This adds to an inherent complexity and uncertainty on psychiatric diagnosis, which relies on the subjective assessment of thoughts, emotions, and behavioral patterns. This uncertainty is evidenced by the recurrent and cyclical revisions and discussions on the fundamental diagnostic tool for such conditions, with its last edition in the DSM-5 [61]. But more importantly for the scope of this work, this poses a potential of including genetically outlying cases in purely genetic studies. This is detrimental to the application of classification algorithms, as those would be trained to learn a genetic background that would not precisely align with the intended phenotype, leading to inconclusive or potentially misleading findings.

The substantial number of samples incorporated into a study can help mitigate this challenge when conducting exploratory and association studies. In fact, it has been suggested that the inclusion of unscreened controls in sufficiently large association studies would not represent a significant impact on its predictive power, provided that the disorders under study present a low prevalence, such is the case for SCZ [17]. Given this, the criteria used for selecting controls in the phs000473.v2.p2 study align with the original research objectives. The criteria for including cases also suit association studies well. Over time, SCZ diagnoses in the Swedish HDR have consistently yielded similar concordance rates, with increased agreement when broader SCZ definitions are considered [16]. A genetic overlap between SCZ and Schizoaffective disorder [62] further support the validity of broader SCZ definitions. This level of agreement confirms the suitability of the selected Swedish HDR cases as a reliable foundation for straightforward association studies. However, whether these suitability concepts are applicable to ML studies remains unexplored.

Preliminary findings from our work reaffirm these associations. Moreover, testing the model on training data validates the relevance of these associations for the dataset in use. Several genes were associated with SCZ in OMIM. Additionally, significantly enriched PANTHER and KEGG pathways, have been linked to SCZ in the literature. However, unlike traditional association studies, ML models are not reliant on individual genotypes, potentially magnifying the impact of misclassified samples on overall results.

Utilizing the original results from the GenNet tool as a benchmark [28], and conducting tests on a selection of variants from the dataset yielded identical findings. Yet, eliminating samples with a higher likelihood of misclassification (Figure 3) significantly improved performance (Table 4), aligning more closely with heritability predictions in the literature [4, 5] and matching the upper bound for the accuracy of a classification model on this dataset, as presented in the estimations from the original GenNet Paper. These results denote a marked influence of outlying samples, at the proportion of reported misdiagnosis rates, on the performance of DL models. It suggests that filtering datasets initially generated for large-scale association studies could enable a more suitable application of ML approaches to complex disease problems. However, the filtering procedures must preserve representativeness to yield the most informative results.

Sample selection essentially defines new sub-cohorts, upon which new comparisons are based. Thus, it expands the utility of case-control studies on complex diseases. By considering the heterogeneity among samples and within the disease itself, studies may be designed to shift the research focus from large-scale findings to more minucious advancements. Given this, these approaches would be both disease and population-specific and it constitutes a step back from generalizable results. However, it approximates complex diseases research to precision medicine, in the sense that sample prioritization, preceding further analyses on smaller and more homogeneous cohorts respective to diseases or populations, would produce increasingly specific results for the samples represented on the study. Given the genetic intricacy of complex diseases, these approaches may be considered and explored to advance its research.

## 5 Conclusions

The present work attempted to adapt the design of a DL study to biological considerations on SCZ. It was verified that reduction of the utilized large-scale case-control dataset to its most representative features maintained a SCZ-driven genetic distinction between cases and controls. Then, DL networks designed upon reference variant-gene annotations detected outlying samples within a case-control dataset on SCZ and verified a considerable influence on the performance of DL models addressing the same dataset. After filtering the outlying samples, the model attained enhanced performances on par with current estimatives for SCZ heritability.

Future efforts should consider developing improved sample prioritization methodologies. Nevertheless, the current results underscore the potential advantages of ML models in complex disease research. However, their application should be tailored to biological issues rather than blindly applied. Non-standard practices, such as testing on training data, may serve as a preliminary step for sample selection, especially in cases of heightened biological and genetic uncertainty, like the example presented in SCZ etiology.

In future endeavors, these models must undergo rigorous testing and validation on additional datasets. Crucially, the training sets should be continually reassessed and updated. The intermediary step introduced here carries the risk of overfitting the model to a specific set of genetic variants and genes, potentially overlooking influential factors not represented in the dataset. Hence, it is imperative to devise novel strategies for executing more judicious sample exclusions.

New data from diverse samples may breathe life into currently insignificant gene-disease associations, necessitating a repetition of the process and an update of the training set and classifier. This approach aligns with the evolving discourse on the diagnosis and etiology of mental health disorders, contributing to a more robust understanding of how differing definitions and diagnostic guidelines for SCZ and related conditions may affect predictability through genomics.
